# A Comparative Study of the Role of Interpersonal Communication, Traditional Media and Social Media in Pro-Environmental Behavior: A China-Based Study

**DOI:** 10.3390/ijerph17061883

**Published:** 2020-03-13

**Authors:** Ruixia Han, Jian Xu

**Affiliations:** 1 School of Media and Communication, Shanghai Jiao Tong University, 800 Dongchuan RD, Minhang District, Shanghai 200240, China; annahan08@sjtu.edu.cn; 2 Institute of Cultural Innovation and Youth Development, Shanghai Jiao Tong University, 800 Dongchuan RD, Minhang District, Shanghai 200240, China; 3 China Institute for Urban Governance, Shanghai Jiao Tong University, 1954 Huashan RD, Xuhui District, Shanghai 200052, China

**Keywords:** interpersonal communication, social media, traditional media, environmental risk perception, willingness to contribute to the environment, pro-environmental behavior, moderating effect

## Abstract

Previous studies have confirmed that information exposure affects pro-environmental behavior. With the rise of social media, new questions emerge in terms of whether different types of information exposure affect pro-environmental behavior differently. Based on a survey of 550 people that was carried out in China, this study aims to compare the different roles of interpersonal communication, traditional media, and social media in affecting the relationships between people’s environmental risk perception, willingness to contribute to the environment, environmental knowledge, environmental concerns, and pro-environmental behavior. Our research discovered that: (1) traditional media has almost no effect on pro-environmental behavior; (2) interpersonal communication can affect pro-environmental behavior through significantly affecting environmental risk perception; (3) social media affects pro-environmental behavior mainly by strengthening the effects of interpersonal communication. The research reveals that while different types of information exposure affect pro-environmental behavior differently, interpersonal communication plays a central role. Concerning the mutual influence between social media and interpersonal communication, we propose that we could promote pro-environmental behavior by activating social media communication.

## 1. Introduction

How to improve pro-environmental behavior is an important topic in many disciplines. In the field of natural resource management, ecology, environmental studies, and sociology, many important factors which influence pro-environmental behavior have been found. For example, Gifford and Nilsson [1] found 18 factors that affect pro-environmental behavior. They include such personal factors as childhood experience, sense of control, values, goals, felt responsibility, and cognitive biases. They also include social factors which include religion, social class, proximity to problematic environmental sites and so on. In addition, in recent years researchers have become increasingly aware of the importance of the mediating variables which function to connect these factors. For example, by conducting a meta-analysis of 57 articles on the influencing factors of pro-environmental behaviors, Bamberg and Möser [2] conclude that there are eight core socio-psychological variables that influence pro-environmental behavior. They include pro-environmental behavioral intention, problem awareness, internal contribution, social norms, feelings of guilt, perceived behavioral control, attitude, and ethical norms. These studies demonstrate that correlations between the different influencing factors of pro-environmental behavior are complicated. It is important to understand what these factors are and what factors play a more important role than others in influencing pro-environmental behavior.

First of all, we find that of all the factors that affect pro-environmental behavior, four types of factors are most prominent. They are environmental risk perception, environmental knowledge, environmental concern, and willingness to contribute to the environment. Stern’s value-belief-norm (VBN) theory states that the perception of environmental problems, awareness of consequences, and assumption of responsibility are the factors that are most closely related to environmental behavior [3]. This demonstrates that environmental risk perception is an important influential variable. The impact of environmental knowledge is also demonstrated in the “environmental knowledge hypothesis” [4] and the “knowledgeable support hypothesis” [5] hypotheses. Studies by Lin and Huang [6], Tseng and Hung [7], and Sapci and Considine [8] all demonstrate the importance of environmental care. However, some studies argue that there is a weak link between environmental concern and pro-environmental behavior [9,10]. Compared to the first three factors, the willingness to contribute to the environment can better measure the sacrifices that people are willing to make to protect the environment, and thus can more accurately predict pro-environmental behavior. At the same time, the willingness to contribute to the environment is also closely related to environmental responsibility. The above four factors are also closely related to the currently popular rational behavior theory [11], planned behavior theory [12], the normative activation theory [13] and VBN theory on pro-environment research. To conclude, they are all important factors that influence pro-environment behavior.

Another important social fact to which we should pay attention to is that the information environment in which people live is undergoing drastic changes. From the earliest interpersonal communication to the emergence of mass media such as radio and television and to the widespread use of the Internet in the past 40 years, the “pseudo-environment” [14] generated by the media is changing. The development of social media in the past 20 years has further accelerated this change. The most important reason is that social media represents both interpersonal and media communication. Take China as an example, social media represented by WeChat is fully intervening in people’s daily lives. Statistics show that 97% of China’s Internet users use WeChat [15]. In recent years, the emerging video social media Tiktok has also spread widely. The question is whether changes in information environment will affect the traditional mode by which key variables influence pro-environment behaviors. Which type of communication has a larger impact on pro-environmental behavior? What are the mechanisms by which different modes of communication affect pro-environmental behavior? Understanding these questions is important to find solutions to promote pro-environmental behavior in specific socio-cultural contexts. To answer these questions, our research focuses on the Chinese public and examines how key factors that influence pro-environmental behavior function differently in interpersonal communication, traditional media, and social media environments.

## 2. Research Theory, Question, and Hypothesis

### 2.1. Pro-Environmental Behavior’s Correlation to Environmental Knowledge, Environmental Concern, Environmental Risk Perception, and Willingness to Contribute to the Environment

Environmental knowledge refers to people’s knowledge about information on environmental change and environmental protection. The “information gap” hypothesis insists that lacking knowledge about environmental damage and methods of environmental protection usually leads to the public’s reluctance to get involved in activities of environmental protection. It is believed that the general popularization of relevant knowledge will help bridge this information gap. Two typical related theories have emerged, namely the “environmental knowledge hypothesis” [12] and “knowledgeable support hypothesis” [13]. In research of the gender difference in pro-environmental behaviors, Blocker and Eckberg [12] discover that people with more environmental knowledge, men and women alike, are more likely to adopt pro-environmental behaviors. This is the logic of the environmental knowledge hypothesis. Davidson and Freudenburg’s study [13] considers the correlation between environmental knowledge and environmental concern. It argues that women’s increasing environmental knowledge does not reduce their environmental concern. Environmental knowledge itself is also an important factor affecting pro-environmental behavior. This is the logic of the “knowledgeable support hypothesis” which focuses on environmental knowledge’s relations to other important variables that affect pro-environmental behavior. Both hypotheses demonstrate that environmental knowledge is an important variable affecting pro-environmental behavior. This importance is further tested by a large number of empirical studies [16,17,18]. Therefore, we propose the following research hypothesis:

**Hypothesis** **1.**  *Environmental knowledge positively relates to pro-environmental behavior*.

In addition to environmental knowledge, environmental concern is another frequently mentioned variable in pro-environmental behavior studies. De Groot believes that environmental concern should be counted as one of the core variables which the planned behavior theory should be concerned with in order to construct pro-environmental behavior models [19]. Many studies have confirmed that environmental concern does affect various behaviors of environmental protection, such as green purchasing behavior [20], fuel consumption behavior [21], energy use [22], etc. In recent years, studies of environmental concern have taken an interdisciplinary turn. For example, Riisgaard et al. believe that environmental concern should be put into the perspective of action-research projects [23]. Examining environmental concern from the perspectives of wealth, gender, and labor should be able to provide a more effective framework for environmental protection. There are also studies that combine environmental concern with the NEP (new environmental paradigm) [24]. In any case, as a variable developed from the 1970s and 1980s, environmental concern has been valued in the study of various types of environmental measurements around the world [25]. Inglehart [26] believes that environmental concern is closely related to post-materialist values. The relationship between environmental concern and pro-environmental behavior, whether directly or through other variables, has also been confirmed by massive research, so we introduce the following research hypothesis:

**Hypothesis** **2.**  *Environmental concern positively relates to pro-environmental behavior*.

Among the various explanations of pro-environmental behavior, cognition-attitude-behavior is the most basic explanation framework (this framework has later been developed into the recognition-affection-attitude-behavior model). It shows that the perception of environmental condition affects people’s pro-environmental behavior, regardless of how this specific mechanism occurs. Among all kinds of environmental awareness, environmental risk perception has become the most direct variable. As early as 1986, in the interpretation framework of Bandura’s social cognitive view of triadic reciprocal causation [27], environmental risk perception has been valued as a cognitive factor influencing environmental events, cognition, emotion, and behavior. Later on, Der-karabetian et al. divided environmental risk perception into two dimensions: cognitive risk perception and emotional risk perception [28]. Among the various specific pro-environmental behaviors, environmental risk perception is considered as an indispensable influencing variable. For example, Keller et al. [29] proposed the Appraisal theory by summing up 40 years of research on risk perception. He believes that this theory is a useful means of integrating cognitive and affective approaches to risk perception. It could promote people’s understanding of the mechanism of how environmental risk perception influences pro-environmental behavior. In any case, the above studies support our next hypothesis:

**Hypothesis** **3.**  *Environmental risk perception positively relates to pro-environmental behavior*.

Rational behavior theory and planned behavior theory are both basic analytical frameworks in the study of pro-environmental behavior. Rational behavior theory emphasizes the importance of self-interest and believes that personal environmental behavior aims to reduce personal health risks. Planned behavior theory considers behavioral intentions as a key variable that affects pro-environmental behaviors and states that multiple factors influence individual behavioral intentions. These two theories focus on people’s consideration of the benefits of pro-environmental behavior, but acquisition of any benefit requires a cost and a price. Therefore, examining people’s environmental contribution and their willingness to sacrifice becomes an important factor to analyze people’s environmental protection behaviors. We have reason to believe that those who are willing to make more sacrifices to environmental protection and improvement are more likely to engage in pro-environmental behaviors. Existing research has usually examined the willingness to contribute to environmental protection in conjunction with environmental responsibility [30] and education is considered to play an important role [31]. Based on the above research we introduce the following assumption:

**Hypothesis** **4.**  *Willingness to contribute to environmental protection positively relates to pro-environmental behavior*.

### 2.2. Interpersonal Influence, Traditional Media, Social Media and Pro-Environmental Behavior

The impact of interpersonal influence on pro-environmental behavior can be traced back to the social learning theory [32], which states that human behavior is a result of continuous reciprocal interaction among cognitive, behavioral, and environmental determinants and that human thoughts, emotions, and behaviors are influenced by both direct experience and indirect acquisition through observation of other people’s behaviors [33]. This theoretical trend is also consistent with Skinner’s operant theory [34] and Bandura’s social cognitive theory [35]. As far as pro-environmental behavior is concerned, Sadachar et al. found that interpersonal influence is a significant indicator of the green consumption behavior among American youths [33], which fully demonstrates the importance of interpersonal communication. From the perspective of pure information transmission, interpersonal communication exerts a more direct influence. For example, Dunlop, Wakefield, & Kashima et al. found that interpersonal discussions on health issues can affect their risk perception and related behaviors [36]. If this influence is generated through information stimulation, deeper interpersonal communication can also affect attitudes and behaviors by changing people’s normative perceptions [19,37]. Research by Nixon and Saphores [38] discovered that people who receive three recycling messages from family and friends are more likely to take this action, and this method of information dissemination initiates a far greater influence than the other methods. Wahlberg and Sjoberg [39] confirm that interpersonal communication holds greater influence than media communication on risk perception. These studies show that interpersonal influence does affect pro-environmental behaviors. It plays a more important role than other sources of information. Therefore, we assume that:

**Hypothesis** **5.**  *Interpersonal influence positively relates to pro-environmental behavior*.

In addition to interpersonal influence, media has also become an important factor in influencing pro-environmental behavior. There are several theoretical perspectives regarding the impact mechanism of media communication on pro-environmental behavior. From the perspective of risk communication, media communication will affect the public’s perception of environmental risks and magnify relevant risk perception [40,41]. Studies have further examined the specific effects of different types of media on the amplification of environmental risk perception. For example, Zeng [42] believes that internet has a greater effect on risk perception amplification than previous media. However, subsequent research has interpreted this relationship in more diverse directions. For example, Wahlberg and Sjoberg [39] believe that media does have an impact on risk perception, but it is not as influential as interpersonal communication, nor is its relationship with behavior change certain. Fischhoff [43] argues that the relationship between media and risk perception should be understood in several stages. In general, it is confirmed that media exposure does affect pro-environmental behavior through influencing people’s risk perception. The second perspective develops from is the uses and satisfaction theory [44]. Media is believed to affect pro-environmental behaviors by improving people’s environmental concerns and environmental information. For example, combining the theories of acculturation and use satisfaction, Holbert et al. [45] pointed out that watching television will increase environmental concern and affect pro-environmental behavior. Trivedi [46] discovered that media affects people’s green purchasing behavior by positively affecting their environmental concern and negatively affecting their internal environmental attitude. Huang’s research of Taiwanese residents [47] discovered that the individual acquisition of global warming information from media will affect their pro-environment behavior. The third perspective analyzes media’s function by combining planned behavior theory with subjective norms theory or media dependence theory. For example, Lee [48] showed that media contact does have a significant impact on adolescents’ pro-environmental behavior in the framework of the attitude-intention-behavior model. Chan’s survey of 173 Hong Kong households [49] confirmed that mass media influences residents’ subjective norms. His research is an effective example of applying planned behavior theory’s analytical model. Similarly, by combining the theory of planned behavior with the theory of media dependence, Ho [50] found the relationship between media dependence and green buying behavior. The theoretical model about mass communication’s impact on pro-environmental behavior is called “the influence of presumed media influence model” (IPMI) [51]. Liao et al. [52] tested the mediating role of perceived media influence between perceived media exposure of others and perceived social norms. In conclusion, studies from all three perspectives show that media could affect people’s environmental behavior.

In fact, media’s influence on pro-environmental behavior is obviously subjected to media’s development stage. In earlier days, the focus was mainly on traditional media’s (such as television, radio, and newspapers) impact on pro-environmental behaviors. The Internet was then included in academic scope. In recent years with the development of social media, people have started to pay attention to its impact. Studies by Ho [50] and Holbert et al. [45] focus on traditional media’s impact on pro-environmental behavior. For example, Holbert [45] discovered that television plays an important mediating role in environmental concern and pro-environmental behavior. Huang’s research [47], which mainly focuses on television, newspapers, and the Internet, suggests that global warming-related information obtained from these three types of media has strongly influenced pro-environmental behaviors. In Ho’s research [50], newspapers and televisions are classified as traditional media compared to the Internet, and their relationships with pro-environmental behavior are analyzed. However, the development of social media in recent years has further expanded the channels and types of information that affect pro-environmental behavior. Likewise, many researchers also acknowledge social media’s potential impact on pro-environmental behavior.

For example, social media has a display function. It enables small behaviors in daily life to be seen and enhances the public’s understanding of their own behavior and the behavior of others, and thus promotes pro-environmental behavior. Social media’s recording function enables people to have an intuitive feeling of their own energy-saving and environmental performance and promote their environmental behavior [53,54]. Human beings’ psychology of social comparison is the important mediator on which social media capitalizes to promote people’s pro-environmental behavior. Hynes [55] directly points out that social media can effectively improve people’s normative cognition by effectively activating people’s psychology of social comparison. A study by Han et al. [56] points out that user-generated content (UGC) is more likely to gain public trust than official information. This information plays an important role in activating pro-environmental regulations, the creation of a pro-environmental online community, and increased public participation in environmental behavior. There is reason to believe that social media is playing a role different from traditional media, including the Internet, by linking media composures to interpersonal contacts. We have the following assumptions that:

**Hypothesis** **6.**  *Traditional media information positively relates to pro-environmental behavior*.

**Hypothesis** **7.**  *Social media information positively relates to pro-environmental behavior*.

### 2.3. Personal Environment-Related Variables, Information Composure, and Pro-Environmental Behavior

The above literature review tells us that environmental knowledge, environmental concern, environmental risk perception, and willingness to contribute to the environment are all important factors affecting pro-environmental behaviors. They constitute individual knowledge and perception variables related to the environment. In other words, most of our perception of the daily environment comes from the “pseudo-environment” created by the media. Many studies [39,50] have shown that even in the age of media communication, interpersonal communication plays a role beyond media communication. The combination of interpersonal with media communication further affects people’s attitudes and behavior. Social media can not only take advantage of interpersonal communication behavior display and information transmission, but also have the characteristics of a wide range of traditional media communication, thus affecting people’s pro-environmental behavior through multiple mechanisms. Our research question is how individual environment-related variables and information exposure variables affect pro-environmental behavior and what are the mutual moderation mechanisms.

Social construction theory provides us with the necessary framework with which to examine the relationship between information exposure, risk perception, and pro-environmental behavior. Nelkin [57] points out that mass media and interpersonal communication play an important role in constructing social risks. Studies by Scherer et al. [58] have tested that “groups or communities of like-minded” can significantly affect people’s risk perception. The occurrence of this effect mechanism is also known as the social contagion effect of risk perception. The analysis by Coleman [59] reveals that interpersonal communication has a greater impact on social risk judgments. These studies support our hypothesis that:

**Hypothesis** **8.**  *Interpersonal communication moderates the relationship between environmental risk perception and pro-environmental behavior*.

With regard to the current media information environment, researchers are more concerned about the impact of media communication on risk perception. For example, Nelkin [57] believes that the mass media are the major vehicle of risk communication. Agha [40] and Mileti [41] further showed that the mass media has a magnifying effect on risk perception. Fischhoff [43] argued that the relationship between media and risk perception should be understood in stages. In sum, these studies show that media plays an important mediating role between risk perception and pro-environmental behavior. At the same time, with the development of media types, current social media also plays an important role in the construction of risk perception. Therefore, we assume that:

**Hypothesis** **9.**  *Traditional media moderates the relationship between environmental risk perception and pro-environmental behavior*.

**Hypothesis** **10.**  *Social media moderates the relationship between environmental risk perception and pro-environmental behavior*.

Information exposure plays a similarly important role between environmental knowledge, environmental concern, willingness to contribute to the environment and pro-environmental behavior, as it does to the relationship between risk perception and pro-environmental behavior. Holbert et al. [45] discovered that watching TV raises environmental concerns and thus promotes pro-environmental behavior. Huang [47] found that individuals’ acquisition of global warming information from television, newspapers, and the Internet could affect their pro-environmental behavior. Chan [49] and Liao et al. [52] both showed that media plays an important role in regulating normative perceptions and environmental behaviors. We have reason to believe that this moderating effect of media does not only exist in a single type of media. Therefore, the moderating effects of different media types and interpersonal communication on pro-environmental behaviors are worth our comparison. We put forward the following assumptions:

**Hypothesis** **11.**  *Interpersonal communication moderates the relationship between environmental concerns and pro-environmental behaviors*.

**Hypothesis** **12.**  *Traditional media moderates the relationship between environmental concern and pro-environmental behavior*.

**Hypothesis** **13.**  *Social media moderates the relationship between environmental concern and pro-environmental behavior*.

**Hypothesis** **14.**  *Interpersonal communication moderates the relationship between environmental knowledge and pro-environmental behavior*.

**Hypothesis** **15.**  *Traditional media moderates the relationship between environmental knowledge and pro-environmental behavior*.

**Hypothesis** **16.**  *Social media moderates the relationship between environmental knowledge and pro-environmental behavior*.

**Hypothesis** **17.**  *Interpersonal communication moderates the relationship between willingness to contribute to the environment and pro-environmental behavior*.

**Hypothesis** **18.**  *Traditional media moderates the relationship between willingness to contribute to the environment and pro-environmental behavior*.

**Hypothesis** **19.**  *Social media moderates the relationship between willingness to contribute to the environment and pro-environmental behavior*.

We all know that social media is different from interpersonal communication and traditional media because it combines the attributes of the two. While social media independently promotes pro-environmental behaviors through its display functions, recording functions, and social comparative stimulus functions, it is still uncertain whether it also moderates the role of traditional media and interpersonal communication in promoting pro-environmental behaviors. The research by Ho [50] points out that traditional media attention and interpersonal communication moderate the impact of media dependence on green purchasing behavior. Wahlberg and Sjoberg [39] have tested that interpersonal communication is more effective than media communication. Regardless of the mechanism of interaction, their research shows that different ways of information exposure impact pro-environmental behavior in a complicated manner. With regard to social media’s combination of both traditional media and interpersonal communication characteristics, we make the following assumptions:

**Hypothesis** **20.**  *Social media moderates the relationship between traditional media and pro-environmental behavior*.

**Hypothesis** **21.**  *Social media moderates the relationship between interpersonal communication and pro-environmental behavior*.

**Figure 1:**
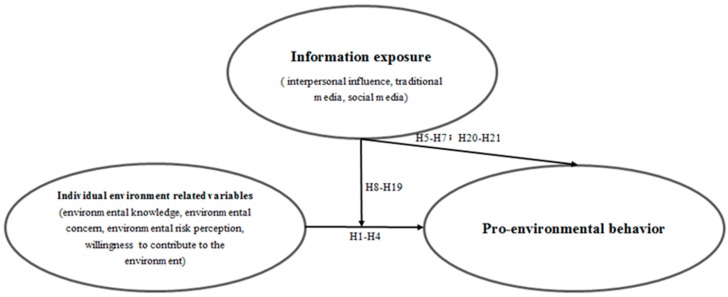
Proposed research model showing the role of information exposure.

The main research models of this study are as follows (Figure 1):

## 3. Research Design

### 3.1. Sample and Data Collection

The data used in this study were obtained through online surveys. Questionnaires were collected between 20–27 August 2019 on China’s large online questionnaire platform (Wenjuanxing: https://www.wjx.cn/). Volunteers were invited to answer questions from the 2.6 million sample database through continuous rolling random distribution. In order to ensure the quality of the recovered data, we adopted the method of IP address setting and content logic design so that each participant could only fill in the questionnaire once. The questionnaire platform randomly sent questionnaires to registered users for multiple rounds until the number of questionnaires reached our requirement. By this method we collected 550 valid questionnaires. The survey covers people from 30 provinces, cities, and autonomous regions in mainland China except Xinjiang (the data platform does not include Xinjiang samples), which can better reflect the relationship between the Chinese public in media use and pro-environmental attitudes and behaviors. Among the recovered samples, 246 were male (44.7%) and 301 were female (55.3%). The education degree distribution was: junior high school and below (1.1%), high school (1.7%), college/university (83.8%), master (11.6%), doctor and above (0.7%). Revenue distribution: <10,000 RMB (10.5%), 10,000–30,000 RMB (8.9%), 30,000–50,000 RMB (8.7%), 50,000–100,000 RMB (33.8%), 100,000–200,000 RMB (30.5%), 200,000–500,000 RMB (6.2%), >500,000 RMB (1.3%). The mean age of the sample population was 30.5 years. The standard deviation of age was 7.683. According to China’s National Bureau of Statistics, the gender ratio of the Chinese population is 104.64 (female 100) [60]. We give weight to female samples so that the overall sample basically represents the gender distribution of the Chinese population. As samples of the survey were mainly from cities, the majority of them belong to the working population with a college degree. The income distribution also basically adheres to the statistical characteristics of the urban residents released by the National Bureau of Statistics of China [61].

### 3.2. Measurement

#### 3.2.1. Pro-Environmental Behavior (PEB)

The dependent variable in this study is the public’s pro-environmental behavior. There are many methods to measure pro-environmental behaviors, such as those developed by Bratt [62], Gatersleben et al. [63], Dono et al. [64], and Tobler et al. [65]. Considering the particularity of the Chinese social and cultural environment, we adopted the measurement items used by Hong [66] in the Chinese General Social Survey (CGSS) in 2003, 2010, and 2015. Related measurement issues can be found in another study we conducted at the same time [67]. The corresponding options in the data file were: never = 0; occasional = 1; often = 2, always = 3. The scale’s internal consistency coefficient Cronbach’s Alpha was 0.741, and the value range of the entire item is 0–30. The mean of this variable was 13.51 and the standard deviation was 3.89.

#### 3.2.2. Measurement of Environment-Related Variables

##### Environmental Concerns (EC)

The environmental concern scale is derived from the Chinese version of the NEP scale [68,69,70], which was tested by Hong [63]. The main problems include (1) the current total population is approaching the limit that the earth can bear; (2) the destruction of nature by human beings often leads to catastrophic consequences; (3) at present, human beings are abusing and destroying the environment; (4) animals and plants have the same right to live as humans; (5) the self-balance ability in nature is strong enough to cope with the impact of a modern industrial society; (6) although humans have special abilities, they are still subject to the laws of nature; (7) that human beings are facing an “environmental crisis” is an exaggerated statement; (8) the Earth is like a spaceship with only limited space and resources; (9) nature. The balance is very fragile and can be easily disrupted; (10) if everything continues as it is, we will soon suffer severe environmental disasters. The corresponding options in the data file were: strongly agree = 2; more agree = 1; general = 0, more disagree = −1, strongly disagree = −2. Among them, 5 and 7 are scored in reverse. The value range of the entire item is from −22 to 22, and Cronbach’s Alpha was 0.714. The mean of this variable was 11.93 and the standard deviation was 3.76.

##### Environmental Knowledge (EK)

The Environmental Knowledge Scale comes from the CGSS2013 version. The questionnaire contains a 10-question environmental knowledge scale to assess the environmental knowledge level of the respondents. Related measurement items can also be found in another of our studies [67]. The overall scale score is 0–10 and Cronbach’s Alpha of this variable was 0.773. The mean of this variable was 9.30 and the standard deviation was 1.23.

##### Environmental Risk Perception (ERP)

The Environmental risk perception scale comes from the CGSS2013 version. Specific measurement issues can be found in Xu and Han’s research [67]. This variable ranges from 0–5 by weighted average. The internal consistency coefficient Cronbach’s Alpha was 0.818. The mean of this variable was 3.74 and the standard deviation was 0.93.

##### Willingness to Contribute to the Environment (WCE)

The scale has been tested by CGSS2013. The corresponding question item in the questionnaire was: “If it is beneficial to environmental governance and protection, would you be willing to engage in the following behaviors?” The corresponding options included: (1) paying a higher price for the goods purchased; (2) paying a higher price in taxes; (3) reducing the convenience of life; (4) reducing the standard of living. Each item was worth 1 point, but none were worth 0 points. The final scoring weighted average value range is 0–4 points. The scale’s internal consistency coefficient, Cronbach’s Alpha, was 0.803. The mean of this variable was 1.97 and the standard deviation was 1.07.

#### 3.2.3. Information Exposure Variables

##### The Influence of Interpersonal Communication (ICI)

The measurement of interpersonal influence refers to the measurement of subjective norms by Park and Smith [71]. The specific measurement item was as follows: “Please express your attitude to the following matters according to the actual situation.” The corresponding options included: (1) my family often does environmental protection things; (2) my friends often do environmental protection things; (3) the daily public environmental protection work of the general public is doing a good job. In the data file, each response was assigned a value of 1–5 according to the frequency of occurrence. The scale’s internal consistency coefficient Cronbach’s Alpha was 0.736. The mean of this variable was 3.33 and the standard deviation was 0.68.

##### Traditional Media Environment Information Exposure (TME) and Social Media Environment Information Exposure (SME)

Considering the use of various media, traditional media mainly includes newspapers, magazines, radio, television, and the Internet. The main types of social media use include WeChat, Weibo, Douyin, Kuaishou, QQ, BaiduTieba, Zhihu, Douban, Facebook, Twitter, and Instagram. Their measurements of obtaining pro-environmental information mainly include four categories: (1) information about environmental change; (2) various discussions about the environment; (3) how other people and places deal with environmental issues; (4) ways and means to protect the environment [67]. We assigned 1 point to each item and obtained the score of each respondent’s environmental related information through traditional media and social media. The final scoring weighted average value range is 0–4 points. The internal consistency coefficient of traditional media environmental information contact scale Cronbach’s Alpha was 0.777, and the internal consistency coefficient of social media environmental information contact scale Cronbach’s Alpha was 0.807. The mean of traditional media environmental information contact scale was 1.80 and the standard deviation was 0.80. The mean of traditional media environmental information contact scale was 1.04 and the standard deviation was 0.60.

#### 3.2.4. Control Variables

The control variables in this study include basic demographic variables. They are gender, age, education (Edu), income, and community participation (CP). Operational measurement of these demographic variables can be referred to in our other study [67].

### 3.3. Data Analysis Methods and Procedures

We used SPSS19.0 software to test the relevant hypotheses using a multi-level regression analysis method. After setting the pro-environment behavior as the dependent variable, we gradually filtered the variables through two steps and performed relevant hypothesis testing. In the first step, we put demographic variables, environment-related variables, and information exposure-related variables into the regression model to examine which of the seven types of variables have a significant effect on pro-environmental behaviors, thereby testing Hypothesis 1–Hypothesis 7. In the second step, we eliminated the non-significant related variables in the test of the regression model (M1-2) in the first step and examined the effects of environment-related variables and the types of information exposure and their cross-variables on pro-environmental behaviors. Four sets of 12 hypotheses (Hypothesis 8–Hypothesis 19) were tested. At the same time, we conducted cross-variable processing of social media, traditional media, and interpersonal communication to examine Hypothesis 20 and Hypothesis 21.

## 4. Findings

### 4.1. Hypothesis Test Results

Table 1 shows the results of a regression equation model that incorporates demographic variables, environment-related variables, and information-exposure related variables, with pro-environmental behavior as the dependent variable. Table 1 (M1-1) shows the results of the regression model using demographic variables and environment-related variables as independent variables. This model mainly shows the influence of each variable on pro-environmental behaviors without information exposure as an introduction. The result shows that environmental knowledge, environmental risk perception, and willingness to contribute to the environment all have a significant impact on pro-environmental behavior, but environmental concern has no significant impact. The model’s explanatory power is 0.238. Table 1 (M1-2) is the result of introducing information-related variables into the model. The results show that environmental knowledge and environmental concern have no significant effect on pro-environmental behaviors, and environmental risk perception and willingness to contribute to the environment have a significant effect. The results of M1-2 support Hypothesis 3 and Hypothesis 4 instead of Hypothesis 1 and Hypothesis 2. Among the variables related to information exposure, interpersonal communication and social media have significant effects on pro-environmental behavior, while traditional media has no significant effect on pro-environmental behavior. This tests Hypothesis 4 and Hypothesis 6 but does not support Hypothesis 5. The explanatory power of the whole model rises to 0.340 and fully illustrates the appropriateness of introducing information exposure variables to explain pro-environmental behavior.

The purpose of Table 2 is to further investigate the moderating effect of information-related variables on the relationship between environmental-related variables and pro-environmental behaviors. M2-1 (Table 2) is the result of removing the insignificant variables from the environment-related variables and information exposure-related variables in Table 1. In this model, it can be seen that the remaining variables include environmental risk perception, willingness to contribute to the environment, interpersonal influence and social media, and their relationships with pro-environmental behaviors are significantly correlated. M2-2 examines interpersonal communication’s moderating effect on environment-related variables and pro-environmental behaviors. It is found that when ICI × ERP and ICI × WCE interaction effects are introduced, the predictive effect of ICI × ERP on pro-environmental behavior is significant, while that of ICI × WCE on pro-environmental behavior is not significant. This indicates that interpersonal communication significantly affects people’s environmental risk perception and pro-environmental behavior, whereas it has no significant impact on the relationship between willingness to contribute to the environment and pro-environmental behavior. M2-3 examines social media’s moderating effect on the relationship between environment-related variables and pro-environmental behaviors. The result shows that with the introduction of such intersecting variables as social media, environmental risk perception, and willingness to contribute to the environment (which are represented as SME × ERP and SME × WCE), the predicting effect of SME × ERP on pro-environmental behavior is significant, while the predicting effect of SME × WCE on pro-environmental behavior is not significant. This indicates that people’s environmental risk perception is also significantly affected by social media, but the willingness to contribute to the environment is not significantly affected by social media.

In order to comparatively examine the moderating effects of interpersonal communication and social media on the relationship between environmental-related variables and pro-environmental behaviors, we put them and their cross-variables with environmental risk perception and willingness to contribute to the environment into the same regression model (M2-4). We discovered that only environmental risk perception and ICI × ERP have a significant predicting effect on pro-environmental behaviors, whereas all other variables are not significant. It confirms that environmental risk perception has a significant effect on pro-environmental behaviors, and interpersonal communication plays an important moderating role between them. Hypothesis 8 was tested. Hypothesis 9–Hypothesis 19 were not tested. As to why Hypothesis 9–Hypothesis 19 have not been tested, we need further research for a detailed analysis.

M2-1 shows that interpersonal communication and social media both have significant effects on pro-environmental behaviors. M2-2 and 2-3 also show that they both moderate the relationship between environmental risk perception and pro-environmental behaviors. Our question is whether they influence each other. As a consequence, we introduce the cross variables of the two into the equation to form M2-5. It is found in the equation that when cross variables are introduced, the predictive effect of social media is no longer significant, whereas the predictive effect of interpersonal communication and the interaction variables are significant. This indicates that social media influences pro-environmental behaviors largely through interpersonal communication. The regression analysis shows that even in the age of social media, interpersonal communication remains the dominating power in influencing pro-environmental behavior, whereas social media mainly functions to facilitate this power of interpersonal communication. Hypothesis 21 was tested.

### 4.2. A Summary of the Testing Results of Our Hypotheses

The main purpose of this article is to examine the role of interpersonal communication, traditional media, and social media in mediating the relationship between environment-related variables and pro-environmental behaviors and to compare their respective mechanisms and effects. With the multiple regression analyses in Table 2 and Table 3, we summarize the testing results of the relevant hypotheses.

It can be seen from Table 3 that (1) among the important environmental-related variables, environmental knowledge and environmental concern have no significant effect on pro-environmental behaviors, while environmental risk perception and willingness to contribute to the environment have a positive correlation with pro-environmental behaviors; (2) among the variables related to information exposure, traditional media information exposure has no significant effect on pro-environmental behaviors, whereas there is a positive correlation between the influence of interpersonal communication, social media information exposure, and pro-environmental behaviors; (3) when placed in the same model and examining the effects of information exposure-related variables on pro-environmental behaviors, it can be found that interpersonal communication has a significant moderating effect on the relationship between environmental risk perception and pro-environmental behaviors, whereas other modes of information exposure do not possess the same significant moderating effects (Hypothesis 9–Hypothesis 19); (4) with regard to social media and interpersonal communication’s influencing mechanism on pro-environmental behaviors, it is confirmed that interpersonal communication has the most significant influencing effect, whereas social media’s effect is only auxiliary.

## 5. Conclusions and Discussion

This study mainly discusses how different modes of information exposure moderate the relationship between environment-related variables and pro-environmental behaviors in the current media environment. The research results show that, compared to interpersonal communication and social media, traditional media has nearly no significant effect. Further investigation of interpersonal communication and social media’s moderating effects on the relationship between environmental-related variables and pro-environmental behaviors demonstrates that both of them have a significant mediating effect on environmental risk perception. However, when compared in the same model, the significant moderating effect of interpersonal communication still exists, whereas social media’s moderating effect is no longer significant. The research also reveals the mechanisms by which interpersonal communication and social media affect pro-environmental behaviors. It shows that the use of social media affects pro-environmental behaviors mainly by strengthening interpersonal communication’s influencing effects. The above research findings have the following theoretical and practical significance.

### 5.1. Theoretical Implications

#### 5.1.1. In the Current Media Environment, the Influence of Traditional Media on Pro-Environmental Behavior is Diminishing and Social Media is Playing a More Important Role than Traditional Media

Most of the existing studies of the influence of media on pro-environmental behavior especially in China focus on traditional media and examine the role of traditional media in moderating the relationship between important environment-related variables and pro-environmental behaviors. For instance, some studies focus on media’s effect on the relationship between environmental risk perception and pro-environmental behavior and put forward the theory of risk amplification. Some studies focus on the role of media in affecting people’s pro-environmental behaviors through enhancing people’s environmental concerns and environmental knowledge. Others analyze media’s influence on pro-environmental behaviors through enhancing people’s value orientation and activating people’s normative perception. Many articles in these studies focus on the traditional media, including the Internet, as the main object of analysis. For example, Holbert’s research [45] focuses on television and Huang’s research [47] focuses on television, newspapers, and the Internet. Chan [49] and Ho [50] also mainly deal with traditional mass media, including the overall theoretical model of IPMI (the influence of presumed media influence) model. Gunther and Storey [51] mainly focus on traditional media, including the Internet. Although the academic community has begun to pay attention to the influence of social media on pro-environmental behavior in recent years, few studies have comparatively analyzed social media and traditional media in the same model. This study demonstrates that social media is playing a more significant role in influencing the relationship between environment-related variables and pro-environmental behaviors. This coincides with the prevailing media suppression/substitution theory [72,73].

#### 5.1.2. Interpersonal Communication Holds the Most Significant Effect on Pro-Environmental Behaviors and Social Media Strengthens This Effect

The influence of interpersonal communication on pro-environmental behavior has long been the focus of many studies. Ho [50] compared respective roles of traditional media and interpersonal communication in media dependence and green purchasing behavior. With many other studies, Ho showed that interpersonal communication plays a greater role than traditional media. Nevertheless, few studies have compared the functions of interpersonal communication and social media in influencing pro-environmental behavior. Our research finds that interpersonal communication still plays a major role in the impact on pro-environmental behavior. Social media as a fusion of interpersonal and media communication further strengthens the role of interpersonal communication. The effects of social media on pro-environmental behavior can also be recognized in other studies. Oakley et al. [53] and Mankoff et al. [54] found that social media puts small behaviors in daily life under public scrutiny and encourages people’s pro-environmental behaviors by improving public understanding of their own behavior and the behavior of others. Social media also affects pro-environmental behaviors with the recording function which helps form public awareness of their deeds and efficiency. Social media also affects pro-environmental behaviors by stimulating people’s psychology of social comparison and improving their recognition of social norms [53]. Han et al. [56] point out that user-generated content (UGC) is more likely to gain public trust than official information by activating pro-environmental norms, creating environmentally friendly online communities, and increasing public pro-environmental participation. Compared to studies which focus exclusively on social media, this study makes a comparative analysis of the mechanism by which interpersonal communication and social media influence pro-environmental behaviors.

### 5.2. Practical Implications

#### 5.2.1. The Dissemination of Pro-Environmental Information and Behavior on Social Media Does Help to Improve People’s Pro-Environmental Behavior

Our research shows that in the current information age, the influence of traditional media on pro-environmental behavior is weakening, while social media is playing a more significant role. A comparison between social media and interpersonal communication’s influencing effects shows that the social media encourages pro-environmental behaviors by moderating interpersonal communication’s influencing effects. Therefore, encouraging people to share their pro-environmental behaviors in daily life on social media is a more effective means than publishing and discussing pro-environmental information on traditional media and on the Internet. In addition to functions of public display, recording, and social comparison, social media also plays an important role in adhering to social norms, guiding social values, and attracting more social trust. Therefore, it is easier to promote the overall social pro-environment behavior by distributing and discussing various types of pro-environmental information on social media, so as to stimulate public participation. In a word, we should value social media’s importance in promoting pro-environmental behaviors. As for whether the decrease in the influence of traditional media is due to reducing concern about environmental issues or other reasons, it is worthy of further research. However, this study at least illustrates the importance of social media dissemination to pro-environmental behavior.

#### 5.2.2. Social Media Has a Greater Impact on People’s Environmental Risk Perception and Willingness to Contribute to the Environment

In this article, we found environmental risk perception and willingness to contribute to the environment as the most important variables among the related variables concerning pro-environmental behaviors. We reconfirm the conclusions of previous studies that increasing environmental awareness, environmental knowledge, and environmental concern can no longer significantly improve public pro-environmental behaviors in China [74,75]. We also discover that, while both interpersonal communication and social media have significant mediating effects on environmental risk perception and willingness to contribute to the environment, interpersonal communication’s mediating effect on environmental risk perception is most significant. This means that interpersonal communication’s demonstration effect is more likely to promote public participation in pro-environmental behaviors. The question is how people should promote this demonstration effect. Our research suggests that we can do this by the dissemination of environmental information and behavioral displays on social media. This tests the assumption that the most direct and effective way to improve the public’s pro-environment behavior in China is by promoting social media’s demonstration effects on pro-environmental behaviors, which in turn sharpens people’s perception of environmental risks and ultimately promotes the overall pro-environmental behavior.

### 5.3. Limitations

This study focuses on examining the roles of interpersonal communication, traditional media, and social media in moderating the relationship between important environment-related factors and pro-environmental behavior. With regard to the conceptualization of the three types of information contact and the selection of important influencing variables, there is much room for further discussion. For example, in the history of information transmission, interpersonal communication happened long before media communication emerged. The classification of traditional media and social media is controversial. According to Ho [50], the Internet is classified as traditional media purely for the reason that social media combines characteristics of both interpersonal and mass media communication. This classification is made simply in order to highlight the particularity of social media. The underlying hypothesis of this classification, as this paper also believes, is social media’s substitution effect on traditional media. However, this hypothesis needs further confirmation. Secondly, this study selects four variables relating to pro-environmental behaviors: environmental concern, environmental knowledge, environmental risk perception, and willingness to contribute to the environment. It shows that environmental concern and environmental knowledge do not have significant impact in the current Chinese context. In effect, there are many discussions about the influencing factors of pro-environmental behavior, and the selection of variables in this study is not complete. For example, Gifford and Nilsson [1] have summarized 18 major personal and social factors of pro-environmental behavior. However, for the analysis and empirical observation of related research on social environmental protection in China, this study only selects the above four variables.

In terms of the research method, this study uses traditional multi-level regression to compare different models. In order to show the development process of the research ideas more clearly, Table 1 mainly shows the basis for our selection of key variables and Table 2 shows the moderating effects of interpersonal communication and social media on environmental contribution willingness and environmental risk perception. M2-4 shows a comprehensive comparison of the two moderating effects. Although this step-by-step process display is beneficial to the multiple displays of the research results of this article, it will cause some confusion for readers. We need to find a better way to present the research results more clearly. At the same time, our research hypothesis is also suitable for the structural equation model, but for the purpose of demonstrating the research process, this research still adopts a multi-level regression analysis method. In addition, in the study of the operationalization of variables, traditional media and social media both use five progressive issues to obtain environmental information, while interpersonal communication mainly uses three issues to measure the effect of demonstration. This difference is mainly based on the understanding of the channels for obtaining environmental information in the current environment and the prerequisite understanding of the effect mechanism of interpersonal communication, but comparing them equally may be worthy of further discussion. Another important issue is that this research is based on a small sample survey based on an online survey platform. Research results will become more meaningful if they are supported by large-scale survey data based on more stringent procedures.

The results of this study show that the impact of traditional media on pro-environmental behavior is not significant. Although this result is based on a relatively small sample, it makes us notice the fact that in an age of widespread social media, the influence of traditional media may be decreasing. Among the important environmental-related influencing factors, interpersonal communication and social media play a significant role in moderating the relationship between environmental risk perceptions and pro-environmental behaviors, on which interpersonal demonstration effect has the greatest influence. Social media can strengthen the moderating effect of the interpersonal demonstration effect. This result echoes studies by Ho [50] and others to a certain extent which insist interpersonal communication still plays an important role in the current era of media integration. It is best to analyze the effect of media in conjunction with interpersonal communication. Social media’s integration of the roles of interpersonal communication and traditional media communication can promote interpersonal effects. This article is an attempt to reveal the mechanism by which different types of information exposure influence pro-environmental behaviors by a small sample survey.

## Figures and Tables

**Table 1:** Multiple regression analysis of main influencing factors of pro-environment behavior (Standard coefficient).

		Zero-Order Correlation	M1-1		M1-2	
*Control variables*			B (SE)	β	B (SE)	β
	Gender	0.014	−0.080 (0.299)	−0.010	−0.089 (0.280)	−0.011
	Age	0.155 ****	0.041 (0.021)	0.080	0.054 (0.020)	0.107 **
	Edu	0.064 ****	0.672 (0.333)	0.079 *	0.449 (0.311)	0.053 *
	Income	0.174 ****	0.162 (0.115)	0.060	0.150 (0.108)	0.055
	CP	0.405 ****	0.468 (0.048)	0.375 ****	0.364 (0.047)	0.292 ****
*Independent variables 1*	EK	−0.108 *	−0.366 (0.128)	−0.116 **	−0.191 (0.121)	−0.060
	EC	0.032	0.018 (0.044)	0.018	0.001 (0.041)	0.001
	ERP	0.172 ****	10.351 (0.115)	0.183 ****	0.920 (0.285)	0.125 ***
	WCE	0.208 ****	0.469 (0.138)	0.130 ***	0.337 (0.130)	0.093 ***
*Independent variables 2*	ICI	0.437 ****			0.566 (0.071)	0.299 ****
	TME	0.276 ****			−0.240 (0.201)	−0.050
	SME	0.250 ****			0.864 (0.270)	0.134 ***
	F		200.102 ****		240.547 ****	
	Adjusted R²		0.238		00.340	

Notes: * *p* < 0.05, ** *p* < 0.01, *** *p* = 0.001, **** *p* = 0.000.

**Table 2:** Multiple regression analysis of the moderating effects of information exposure on pro-environmental behavior (standard coefficient).

		Zero-Order Correlation	M2-1		M2-2		M2-3		M2-4		M2-5	
			B(SE)	β	B(SE)	β	B(SE)	β	B(SE)	β	B(SE)	β
*Control variables 1*	Age	0.155 ****	0.067 (0.018)	0.133 ****	0.061 (0.018)	0.121 ***	0.065 (0.019)	0.128 ***	0.065 (0.019)	0.128 ****	0.063 (0.018)	0.125 ****
	Edu	00.064	0.452 (0.297)	0.053	0.485 (0.296)	0.057	0.652 (0.314)	0.077 *	0.494 (0.294)	0.058	0.453 (0.296)	0.053
	CP	0.405 ****	0.379 (0.046)	0.303 ****	0.403 (0.045)	0.322 ****	0.441 (0.048)	0.353 ****	0.379 (0.045)	0.303 ****	0.381 (0.045)	0.305 ****
*Control variables 2*	ERP	0.172 ****	0.782 (0.262)	0.106 **	−30.046 (10.091)	−0.413 **	0.016 (0.533)	0.002	−30.052 (10.092)	−0.414 **	0.739 (0.262)	0.100 **
	WCE	0.208 **	0.318 (0.129)	0.088 *	0.470 (0.612)	0.130	0.582 (0.263)	0.161 *	0.515 (0.612)	0.142	0.337 (0.128)	0.093 **
	ICI	0.437 ****	0.589 (0.070)	0.311 ****	−0.827 (0.432)	−0.437			−0.631 (0.457)	−0.334	0.346 (0.120)	0.183 **
	SME	0.250 ****	0.679 (0.236)	0.105 **			−20.679 (10.718)	−0.415	−10.355 (10.714)	−0.210	−10.904 (10.064)	−0.295
*Interaction variables*	ICI × ERP	0.440 ****			0.394 (00.108)	10.024 ****			0.329 (0.114)	0.856 **		
	ICI × WCE	0.320 ****			−0.017 (0.059)	−0.053			−0.012 (0.063)	0.038		
	SME × ERP	0.289 ****					10.020 (0.421)	0.642 *	0.556 (0.418)	0.350		
	SME × WCE	0.273 ****					−0.111 (0.214)	−0.053	−00.079 (0.214)	−0.037		
	ICI × SME	0.360 ****									0.245 (0.099)	0.456 *
	F		40.885 ****		36.702 ****		24.910 ****		27.833 ****		36.982 ****	
	Adjusted R²		0.337		0.342		0.258		0.350		0.343	

Notes: * *p* < 0.05, ** *p* < 0.01, *** *p* = 0. 001, **** *p* = 0.000.

**Table 3:** A summary of test results.

	Aim	Hypothesis		Model	Result
1	Test on the relationship between environment-related variables and pro-environmental behavior	H1	EK → PEB	M1-2	×
		H2	EC → PEB	M1-2	×
		H3	ERP → PEB	M1-2	√
		H4	WCE → PEB	M1-2	√
2	Tests on the relationship between information exposure variables and pro-environmental behavior	H5	ICI → PEB	M1-2	√
		H6	TME → PEB	M1-2	√
		H7	SME → PEB	M1-2	√
3	Tests on the moderating effects of different types of information composure on the relationship between environmental related variables and pro-environmental behaviors	H8	ICI × ERP → PEB	M2-4	√
		H9	TME × ERP → PEB	M2-4	×
		H10	SME × ERP → PEB	M2-4	×
		H11	ICI × EK → PEB	×	×
		H12	TME × EK → PEB	×	×
		H13	SME × EK → PEB	×	×
		H14	ICI × EC → PEB	×	×
		H15	TME × EC → PEB	×	×
		H16	SME × EC → PEB	×	×
		H17	ICI × WCE → PEB	M2-4	×
		H18	TME × WCE → PEB	M2-4	×
		H19	SME × WCE → PEB	M2-4	×
4	Moderating role of social media in the relationship between interpersonal communication, traditional media and pro-environmental behavior	H20	TME × SME → PEB	×	×
		H21	ICI × SME → PEB	M2-5	√
